# Mechanisms of action of molecules with anti-TNF-alpha activity on intestinal barrier inflammation: A systematic review protocol

**DOI:** 10.1097/MD.0000000000017285

**Published:** 2019-09-27

**Authors:** Mayara Santa Rosa Lima, Vanessa Cristina Oliveira de Lima, Grasiela Piuvezam, Kesley Pablo Morais de Azevedo, Bruna Leal Lima Maciel, Ana Heloneida de Araújo Morais

**Affiliations:** a Biochemistry Postgraduate Program, Biosciences Center; b Collective Health Postgraduate Program (PPGSCoL); c Nutrition Postgraduate Program, Center for Health Sciences, Federal University of Rio Grande do Norte, Natal, RN, Brazil.

**Keywords:** anti-inflammatory agents, intestinal mucosa, systematic review, tumor necrosis factor-alpha

## Abstract

**Background:**

Tumor necrosis factor-alpha (TNF-alpha), among cytokines that mediate the inflammatory process, plays an important role in diseases involving the loss of intestinal barrier integrity. Several molecules with anti-TNF-alpha activity have been studied aiming to develop new therapies. The purpose of this paper is to describe the systematic review protocol of experimental studies that determine mechanisms of action of molecules with anti-TNF-alpha activity on intestinal barrier inflammation.

**Methods:**

This protocol is guided by the Preferred Reporting Items for Systematic Reviews and Meta-Analyzes Protocols (PRISMA-P). The databases to be searched are PubMed, EMBASE, Scopus, ScienceDirect, and Web of Science. Experimental studies in rats or mice that assessed the activity of anti-TNF-alpha molecules in models of intestinal barrier inflammation will be included in the systematic review. Studies characteristics, experimental model, and main results will be described and the bias risk assessment will be performed. Two independent reviewers will perform study selection, data extraction, and methodological quality assessment. A narrative synthesis will be made for the included studies. Also, if sufficient data is available, a meta-analysis will be conducted. *I*^2^ statistics will be used to assess heterogeneity.

**Results:**

The present protocol will assist in producing a systematic review that identifies the mechanisms underlying the reduction of TNF-alpha in intestinal barrier inflammation models.

**Conclusion:**

The systematic review may contribute to the theoretical basis of research on new molecules with anti-TNF-alpha potential and, consequently, in the development of new therapies employed in humans.

**PROSPERO registration number:**

CRD42019131862.

## 1 Introduction

The gastrointestinal mucosa separates internal from external environment, and allows only small antigens and microorganisms amounts cross the epithelium, preventing the passage of potentially harmful substances. This ability to protect the body from damage from the luminal content and mucosal permeability control constitutes the intestinal barrier function.^[1]^

Normal functioning of the intestinal barrier is fundamental for homeostasis, while the disruption of barrier mechanisms leads to increased mucosal permeability to luminal antigens and/or microorganisms that cross the intestinal epithelium and potentially induce epithelial-neuroimmune disorders that facilitate the gut inflammation development.^[2]^ Changes in barrier function have been widely implicated in the origin and development of many digestive diseases such as celiac disease, inflammatory bowel disease, irritable bowel syndrome and food allergies; and non-digestive, such as schizophrenia, diabetes, and sepsis.^[3]^

Tumor necrosis factor-alpha (TNF-alpha) has been widely studied and reviewed in the scientific literature regarding its participation in mechanisms involving inflammation-related cell pathways in the intestinal barrier.^[4]^

TNF-alpha is a cytokine produced by several cell types; however, the main producers are monocytic lineage cells, such as macrophages. This cytokine plays a key role during stationary or pathological conditions, for example, infections, lesions, inflammation, and tumor development.^[5]^

Once released from macrophages, which constitute the first line of defense, TNF-alpha activates other immune cells and mediates the production of additional proinflammatory cytokines during inflammatory responses.^[6]^ TNF-alpha also has a direct impact on the intestinal epithelial barrier, since it directly disrupts the intestinal tight junctions.^[7,8]^

In this perspective, several studies aim to promote the protection or recovery of intestinal barrier integrity and functionality through substances with anti-TNF-alpha activity. Understanding how these molecules act is essential for the development of new drugs with more specific action to combat inflammation in the gut. Therefore, studies are needed to discuss the mechanisms of action of molecules with anti-TNF-alpha activity on the intestinal barrier.

When it comes to understanding the mechanisms of action of these substances, most studies are developed in animal models, mainly mice and rats, used in many fundamental investigations for understanding human organism physiology, as well as for the development of new medical therapies.^[9]^ Prior to clinical application several treatments are preceded by animal experiments. These experiments are important for studying the efficacy and/or safety of interventions for humans. In this sense, awareness has increased about the importance of conducting systematic reviews in the field of laboratory animal experimentation.^[10]^ We found recent examples of systematic reviews of animal studies^[11,12]^ and systematic review protocols on animal therapies and interventions.^[13,14]^

Thus, the aim of this paper is to describe the systematic review protocol with experimental studies that determine mechanisms of action of molecules with anti-TNF-alpha activity on intestinal barrier inflammation.

## 2 Methods

### 2.1 Protocol and registration

This protocol has been prepared according to the guidelines described in Preferred Reporting Items for Systematic Reviews and Meta-Analyses Protocols (PRISMA-P).^[15]^ A 17-item checklist was used to improve the quality of the systematic review data.

The protocol was registered with the International Prospective Register of Systematic Reviews (PROSPERO) on May 24, 2019 (CRD42019131862), and is available at: http://www.crd.york.ac.uk/PROSPERO/display_record.php?ID=CRD42019131862.

### 2.2 Eligibility criteria

Peer-reviewed journal articles that meet eligibility criteria based on study population, interventions, control, and outcomes (PICOS)^[16]^ will be included in the review.

#### 2.2.1 Inclusion criteria

The review will include original articles resulting from experimental studies carried out in rats or mice of both sexes, without water or diet restriction, with diagnosis of intestine inflammation at the beginning of the experiment; studies evaluating therapy with anti-TNF-alpha molecule and the effect of this treatment on the intestinal barrier (protection/recovery/damage or lack of effect); studies with intestinal barrier cells of rats or mice that evaluated the same treatment and measured the same outcomes previously cited.

#### 2.2.2 Exclusion criteria

Review articles, case reports, comments, editorials, letters to the editor, theses, conference proceedings will be excluded; studies with other animal/cell models; studies evaluating anti-TNF-alpha treatment in other inflammatory diseases; studies that do not present at least one measure of TNF-alpha reduction or its activity; studies that do not address mechanisms of action of the molecules studied to obtain the effects found.

### 2.3 Information sources and literature search

In the identification phase of the studies, search strategies will be developed based on keywords indexed in the Medical Subject Headings (MeSH). Equations will be used with combinations of descriptors related to intestinal barrier, inflammation, and intervention using anti-TNF-alpha agents, accompanied by the boolean operator AND. The search strategies will be applied to the following electronic databases: PubMed, Scopus, Web of Science (WOS), Excerpta Medica Database (EMBASE) and Science Direct (Table 1).

**Table 1:**
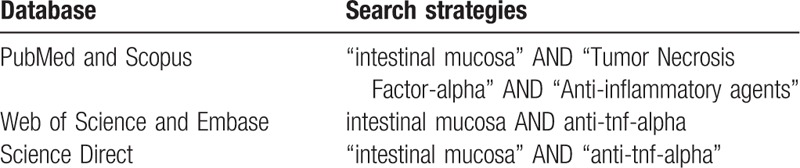
Search strategies for each database.

Database searches will be performed independently by 2 researchers. Initial searches will test preliminary equations with the prospect of applying highly sensitive search strategies. Articles will be imported into Mendeley reference manager (1.17.11) and duplicates will be deleted.

**Figure 1:**
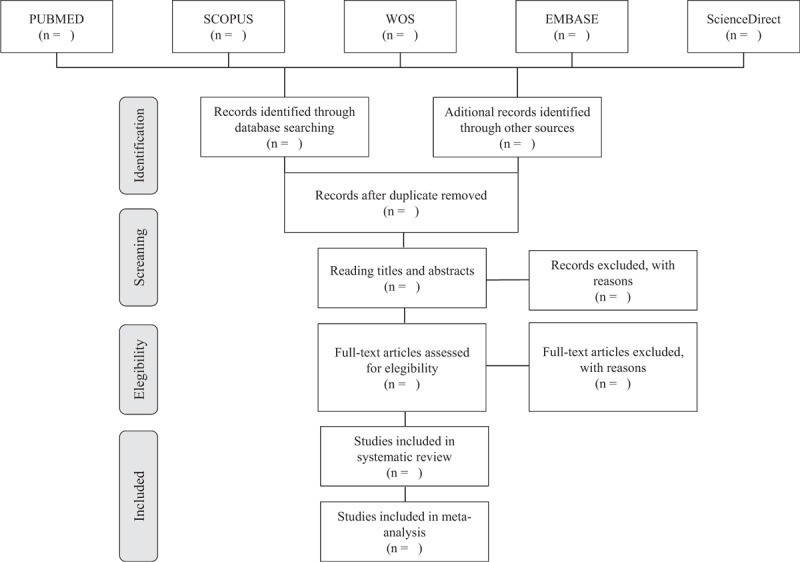
Article selection flowchart. Adapted from PRISMA-P.^[15]^ PRISMA-P = Preferred Reporting Items for Systematic Reviews and Meta-Analyses Protocols.

The initial evaluation of the studies will be carried out by 2 reviewers independently by reading titles and abstracts, following the eligibility criteria. Then, the full text of the articles will be analyzed and studies that meet the inclusion criteria of the systematic review will be selected (Fig. 1). Disagreements that occur during the screening phases will be solved with the assistance of a third reviewer. The researchers will review the full text of all studies considered eligible for inclusion for analysis. The references of the included articles will also be reviewed to identify those potentially eligible studies not found in the database search, considered as manual search.

### 2.4 Data extraction

Data extraction will be done in a standardized manner by 2 researchers independently, using a spreadsheet prepared in the Microsoft Excel program. The following information will be entered in the spreadsheet: first author; year of publication; groups; number of animals per group; species; sex; average weight; average age; diet; way to induce inflammation in the intestinal barrier; type of molecule administered; quantity, time, frequency, route and vehicle of administration; dosage of TNF-alpha (serum, gene, or protein expression, etc); effects of treatment on the intestinal barrier (expression of tight junction proteins, intestinal permeability evaluation, epithelial damage score, histological analysis, etc); mechanisms discussed for the effects found; and other important information.

For any relevant data that is missing in the manuscripts, contact with the study authors will be attempted. If the required information is not obtained, the data will be excluded from analysis and addressed in the discussion section.

### 2.5 Risk of bias assessment

Two reviewers will assess the risk of bias in the selected studies and differences, if any, will be solved by consulting a third reviewer. For the assessment of risk of bias the SYstematic Review Center for Laboratory animal Experimentation (SYRCLE) tool will be used.^[17]^ Reviewers will be previously trained and calibrated to ensure uniformity in criteria evaluation.

### 2.6 Data analysis and synthesis

A narrative synthesis of the included studies will be made. Results corresponding to the interventions effects will be described and will consider the difference in means and *P* values for the measurements assessed before and after the intervention. Comparative analyses performed between intervention groups and between intervention and control groups will be presented.

Heterogeneity between study results will be assessed using a standard chi-squared test with a significance level of 0.05. To assess heterogeneity, we plan to calculate the *I*^2^ statistic, which is a quantitative measure of inconsistency between studies. A value of 0% indicates no heterogeneity was observed, while *I*^2^ values of 50% indicate a substantial level of heterogeneity. If possible, funnel plots will be used to assess the presence of potential reporting biases. A linear regression approach will be used to evaluate funnel plot asymmetry.

## 3 Discussion

Studies about therapies that can help in prevention and treatment of diseases that affect the intestinal barrier integrity are relevant, as many of them present major challenges for the world's health-care systems. A systematic review study about the worldwide incidence and prevalence of inflammatory bowel disease highlighted that in the early 21st century these diseases became a global health problem with increasing incidence in newly industrialized countries in Asia, South America, and Africa, where societies have become more westernized. Regarding the estimated prevalence, although low in the mentioned countries, it continues to increase in North America, Oceania, and many European countries, surpassing 0.3%.^[18]^

In a systematic review by Pedersen et al^[19]^ regarding the important pathways for the management of inflammatory bowel diseases, the authors highlight that TNF-alpha plays a key role in the inflammatory cascade that orchestrates chronic intestinal inflammation, pointing that anti-TNF-alpha agents are the most effective anti-cytokine treatment for these diseases.

In this perspective, some therapies for intestinal diseases mainly involve the administration of anti-TNF-alpha monoclonal antibodies^[20]^ in conjunction with other broadly acting adjuvants such as corticosteroids, prebiotics, and probiotics.^[21]^ The literature presents several review studies that address the use of antibodies in anti-TNF-alpha therapy, especially in cases of inflammatory bowel disease.^[22–26]^

However, it is observed that a significant proportion of patients do not respond to induction therapy with antibodies or become intolerant due to loss of treatment effect over time. This therapy is also associated with the risk of infusion reactions.^[19]^ In addition, some patients do not meet eligibility criteria for antibody therapy, such as those diagnosed with active or latent tuberculosis, HIV, hepatitis B, or other active infections. Patients with a history of lymphoproliferative disorders, severe congestive heart failure, or demyelinating disease also should not be treated with this therapy.^[27]^ In this sense, the need to identify new treatment options for these diseases becomes urgent.

Due to limitations of classical therapies for the treatment of diseases in which TNF-alpha is involved in the disruption of intestinal barrier integrity mechanisms, many alternative molecules have been evaluated in order to develop new drugs.^[28]^ Curcumin derivatives,^[29–31]^ epicatechin,^[32]^ short-chain fatty acids,^[33]^ enzyme inhibitors,^[34]^ and even well-established drugs for other treatments^[35]^ have been employed to reduce the inflammatory process by reducing TNF-alpha, with effects on improving epithelial mucosal integrity.

Since such molecules are diverse, the question is how different structures achieve a common result. Database searches indicated the absence of systematic reviews that group the main mechanisms of action of molecules whose therapeutic proposal focuses on the reduction of TNF-alpha or its activity. Thus, the present protocol will assist in producing a systematic review that identifies the mechanisms underlying these effects in intestinal barrier inflammation models and may contribute to the theoretical basis of research on new molecules with anti-TNF-alpha potential and, consequently, in the development of new therapies employed in humans.

## Acknowledgments

The authors thank the Coordination for the Improvement of Higher Education Personnel (CAPES) for the incentive by granting PhD scholarships, and the National Council for Scientific and Technological Development (Award Number: 426116/2018-6 - CNPq).

## Author contributions

**Conceptualization:** Mayara Santa Rosa Lima, Vanessa Cristina Oliveira de Lima Grasiela Piuvezam, Ana Heloneida de Araújo Morais.

**Formal analysis:** Grasiela Piuvezam, Bruna Leal Lima Maciel, Ana Heloneida de Araújo Morais.

**Funding acquisition:** Ana Heloneida de Araújo Morais.

**Investigation:** Mayara Santa Rosa Lima, Vanessa Cristina Oliveira de Lima, Grasiela Piuvezam, Kesley Pablo Morais de Azevedo.

**Methodology**: Mayara Santa Rosa Lima, Vanessa Cristina Oliveira de Lima, Grasiela Piuvezam, Kesley Pablo Morais de Azevedo.

**Supervision:** Grasiela Piuvezam, Ana Heloneida de Araújo Morais.

**Writing – original draft:** Mayara Santa Rosa Lima, Vanessa Cristina Oliveira de Lima.

**Writing – review & editing:** Grasiela Piuvezam, Kesley Pablo Morais de Azevedo, Bruna Leal Lima Maciel, Ana Heloneida de Araújo Morais.

Mayara Santa Rosa Lima orcid: 0000-0003-3006-7700.

Vanessa Cristina Oliveira de Lima orcid: 0000-0002-5709-3805.

Grasiela Piuvezam orcid: 0000-0002-2343-7251.

Kesley Pablo Morais de Azevedo orcid: 0000-0002-7849-2661.

Bruna Leal Lima Maciel orcid: 0000-0002-0724-1961.

Ana Heloneida de Araújo Morais orcid: 0000-0002-6460-911X.

## References

[1] Bischoff SC Barbara G Buurman W Intestinal permeability - a new target for disease prevention and therapy. BMC Gastroenterol 2014;14:189. 25407511 10.1186/s12876-014-0189-7 PMC4253991

[2] Albert-Bayo M Paracuellos C González-Castro AM Intestinal mucosal mast cells: key modulators of barrier function and homeostasis. Cells 2019;8:pii: E135. 30744042 10.3390/cells8020135 PMC6407111

[3] Konig J Wells J Cani PD Human intestinal barrier function in health and disease. Clin Transl Gastroenterol 2016;7:e196. 27763627 10.1038/ctg.2016.54 PMC5288588

[4] Billmeier U Dieterich W Neurath MF Molecular mechanism of action of anti-tumor necrosis factor antibodies in inflammatory bowel diseases. World J Gastroenterol 2016;22:9300–13. 27895418 10.3748/wjg.v22.i42.9300 PMC5107694

[5] Ruder B Atreya R Becker C Tumour necrosis factor alpha in intestinal homeostasis and gut related diseases. Int J Mol Sci 2019;20:pii: E1887. 30995806 10.3390/ijms20081887 PMC6515381

[6] Cook AD Christensen AD Tewari D Immune cytokines and their receptors in inflammatory pain. Trends Immunol 2018;39:240–55. 29338939 10.1016/j.it.2017.12.003

[7] Ye D Ma I Ma TY Molecular mechanism of tumor necrosis factor-α modulation of intestinal epithelial tight junction barrier. Am J Physiol Gastrointest Liver Physiol 2006;290:G496–504. 16474009 10.1152/ajpgi.00318.2005

[8] Al-Sadi R Guo S Ye D TNF-α Modulation of intestinal epithelial tight junction barrier is regulated by ERK1/2 activation of Elk-1. Am J Pathol 2013;183:1871–84. 24121020 10.1016/j.ajpath.2013.09.001 PMC5745548

[9] DaMatta RA Animal models in biomedical research [Portuguese]. Sci Med 2010;20:210–1.

[10] Hooijmans CR Rovers M de Vries RB An initiative to facilitate well-informed decision-making in laboratory animal research: report of the First International Symposium on Systematic Reviews in Laboratory Animal Science. Lab Anim 2012;46:356–7. 22969143 10.1258/la.2012.012052

[11] Crean AJ Senior AM High-fat diets reduce male reproductive success in animal models: a systematic review and meta-analysis. Obes Rev 2019;20:921–33. 30756459 10.1111/obr.12827

[12] Hooijmans CR Hlavica M Schuler FAF Remyelination promoting therapies in multiple sclerosis animal models: a systematic review and meta-analysis. Sci Rep 2019;9:822. 30696832 10.1038/s41598-018-35734-4 PMC6351564

[13] Zhang Y Yu HJ Shi SZ Effects of different interventions on animal models of ischemic stroke: protocol for an overview and a network meta-analysis. Medicine (Baltimore) 2019;98:e15384. 31027130 10.1097/MD.0000000000015384 PMC6831226

[14] Chorath KT Willis MJ Morton-Gonzaba N Mesenchymal stem cells for sensorineural hearing loss: protocol for a systematic review of preclinical studies. Syst Rev 2019;8:126. 31128597 10.1186/s13643-019-1015-7 PMC6535185

[15] Moher D Shamseer L Clarke M Preferred reporting items for systematic review and meta-analysis protocols (PRISMA-P) 2015 statement. Syst Rev 2015;4:1. 25554246 10.1186/2046-4053-4-1 PMC4320440

[16] Huang X Lin J Demner-Fushman D Evaluation of PICO as a knowledge representation for clinical questions. AMIA Annu Symp Proc 2006;2006:359–63. PMC1839740 17238363

[17] Hooijmans CR Rovers MM de Vries RBM SYRCLE's risk of bias tool for animal studies. BMC Med Res Methodol 2014;14:43. 24667063 10.1186/1471-2288-14-43 PMC4230647

[18] Ng SC Shi HY Hamidi N Worldwide incidence and prevalence of inflammatory bowel disease in the 21st century: a systematic review of population-based studies. Lancet 2017;390:2769–78. 29050646 10.1016/S0140-6736(17)32448-0

[19] Pedersen J Coskun M Soendergaard C Inflammatory pathways of importance for management of inflammatory bowel disease. World J Gastroenterol 2014;20:64–77. 24415859 10.3748/wjg.v20.i1.64 PMC3886034

[20] Eberhardson M Hedin CRH Carlson M Towards improved control of inflammatory bowel disease. Scand J Immunol 2019;89:e12745. 30582196 10.1111/sji.12745

[21] Sales-Campos H Basso PJ Alves VBF Classical and recent advances in the treatment of inflammatory bowel diseases. Braz J Med Biol Res 2015;48:96–107. 25466162 10.1590/1414-431X20143774 PMC4321214

[22] Behm BW Bickston SJ Tumor necrosis factor-alpha antibody for maintenance of remission in Crohn's disease. Cochrane Database Syst Rev 2008;CD006893. 18254120 10.1002/14651858.CD006893

[23] Huang X Lv B Jin H A meta-analysis of the therapeutic effects of tumor necrosis factor-α blockers on ulcerative colitis. Eur J Clin Pharmacol 2011;67:759–66. 21691804 10.1007/s00228-011-1079-3

[24] Chaparro M Guerra I Muñoz-Linares P Systematic review: antibodies and anti-TNF-α levels in inflammatory bowel disease. Aliment Pharmacol Ther 2012;35:971–86. 22443153 10.1111/j.1365-2036.2012.05057.x

[25] Kawalec P Mikrut A Wiśniewska N Tumor necrosis factor-α antibodies (infliximab, adalimumab and certolizumab) in Crohn's disease: systematic review and meta-analysis. Arch Med Sci 2013;9:765–79. 24273556 10.5114/aoms.2013.38670 PMC3832823

[26] Slevin SM Egan LJ New insights into the mechanisms of action of anti-tumor necrosis factor-α monoclonal antibodies in inflammatory bowel disease. Inflamm Bowel Dis 2015;21:2909–20. 26348448 10.1097/MIB.0000000000000533

[27] Billiet T Rutgeerts P Ferrante M Targeting TNF-α for the treatment of inflammatory bowel disease. Expert Opin Biol Ther 2014;14:75–101. 24206084 10.1517/14712598.2014.858695

[28] Giuffrida P Cococcia S Delliponti M Controlling gut inflammation by restoring anti-inflammatory pathways in inflammatory bowel disease. Cells 2019;8:pii: E397. 31052214 10.3390/cells8050397 PMC6562982

[29] Arafa HM Hemeida RA El-Bahrawy AI Prophylactic role of curcumin in dextran sulfate sodium (DSS)-induced ulcerative colitis murine model. Food Chem Toxicol 2009;47:1311–7. 19285535 10.1016/j.fct.2009.03.003

[30] Song WB Wang YY Meng FS New Curcumin protects intestinal mucosal barrier function of rat enteritis via activation of MKP-1 and attenuation of p38 and NF-κB activation. PLoS One 2010;5:e12969. 20885979 10.1371/journal.pone.0012969 PMC2945766

[31] Li CP Li JH He SY Effect of curcumin on p38MAPK expression in DSS-induced murine ulcerative colitis. Genet Mol Res 2015;14:3450–8. 25966111 10.4238/2015.April.15.8

[32] Cremonini E Wang Z Bettaieb A (-)-Epicatechin protects the intestinal barrier from high fat diet-induced permeabilization: implications for steatosis and insulin resistance. Redox Biol 2018;14:588–99. 29154190 10.1016/j.redox.2017.11.002 PMC5691220

[33] Matheus VA Monteiro LCS Oliveira RB Butyrate reduces high-fat diet-induced metabolic alterations, hepatic steatosis and pancreatic beta cell and intestinal barrier dysfunctions in prediabetic mice. Exp Biol Med 2017;242:1214–26. 10.1177/1535370217708188 PMC5476343 28504618

[34] Cho EY Choi SC Lee SH Nafamostat mesilate attenuates colonic inflammation and mast cell infiltration in the experimental colitis. Int Immunopharmacol 2011;11:412–7. 21187179 10.1016/j.intimp.2010.12.008

[35] Lee JY Kim JS Kim JM Simvastatin inhibits NF-κB signaling in intestinal epithelial cells and ameliorates acute murine colitis. Int Immunopharmacol 2007;7:241–8. 17178392 10.1016/j.intimp.2006.10.013

